# The Effect of Surrounding Vegetation on the Mycorrhizal Fungal Communities of the Temperate Tree *Crataegus monogyna* Jacq.

**DOI:** 10.3389/ffunb.2021.741813

**Published:** 2021-10-25

**Authors:** Margaux Boeraeve, Olivier Leroux, Ruben De Lange, Annemieke Verbeken, Hans Jacquemyn

**Affiliations:** ^1^Plant Conservation and Population Biology, Biology Department, KU Leuven, Leuven, Belgium; ^2^Department of Biology, Ghent University, Ghent, Belgium; ^3^Research Group Mycology, Department of Biology, Ghent University, Ghent, Belgium

**Keywords:** dual-mycorrhizal, ectomycorrhizal, arbuscular mycorrhiza, WGA-FITC, metabarcoding

## Abstract

About 90% of all land plants form mycorrhiza to facilitate the acquisition of essential nutrients such as phosphorus, nitrogen, and sometimes carbon. Based on the morphology of the interaction and the identity of the interacting plants and fungi, four major mycorrhizal types have been distinguished: arbuscular mycorrhiza (AM), ectomycorrhizal (EcM), ericoid mycorrhiza, and orchid mycorrhiza. Although most plants are assumed to form only one type of mycorrhiza, some species simultaneously form associations with two mycorrhizal types within a single root system. However, the dual-mycorrhizal status of many species is under discussion and in some plant species the simultaneous association with two mycorrhizal types varies in space or time or depends on the ecological context. Here, we assessed the mycorrhizal communities associating with common hawthorn (*Crataegus monogyna*), a small tree that commonly associates with AM fungi, and investigated the potential factors that underlie variation in mycorrhizal community composition. Histological staining of *C. monogyna* roots showed the presence of a Hartig net and hyphal sheaths in and around the roots, demonstrating the capacity of *C. monogyna* to form EcM. Meta-barcoding of soil and root samples of *C. monogyna* collected in AM-dominated grassland vegetation and in mixed AM + EcM forest vegetation showed a much higher number of EcM sequences and OTUs in root and soil samples from mixed AM + EcM vegetation than in samples from pure AM vegetation. We conclude that *C. monogyna* is able to form both AM and EcM, but that the extent to which it does depends on the environmental context, i.e., the mycorrhizal type of the surrounding vegetation.

## Introduction

With more than 90% of the land plants worldwide forming mycorrhiza, this is the ecologically most important mutualistic association between fungi and plants (Smith and Read, 2008; van der Heijden et al., 2015; Brundrett and Tedersoo, 2018). In return for carbohydrates, the fungus increases nutrient and water uptake by the plant and can provide protection against pests and pathogens (Marx, 1972; Smith and Read, 2008; Cameron et al., 2013). Depending on the morphology of the roots and the taxonomic plant and fungal groups involved, four main types of mycorrhiza can be distinguished: arbuscular mycorrhiza (AM), ectomycorrhizal (EcM), ericoid mycorrhiza and orchid mycorrhiza (van der Heijden et al., 2015; Brundrett and Tedersoo, 2018). Although most plants are assumed to form only one type of mycorrhiza (Soudzilovskaia et al., 2020), some species are known to form associations with fungi of more than one mycorrhizal type (Teste et al., 2020). These plants are referred to as dual-mycorrhizal plants and generally form AM and EcM (Teste et al., 2020).

With 71% of all land plants consistently (and 7% inconsistently) associating with fungi of the phylum Glomeromycota, AM is the most widespread type of mycorrhiza (Brundrett and Tedersoo, 2018). This mycorrhizal type can be further subdivided in two morphological types: the *Arum*-type, characterized by the presence of arbuscules and intercellular hyphae, and the *Paris*-type, characterized by the presence of intracellular hyphal coils (Dickson et al., 2007). About 8,500 plant species are known to form associations with ectomycorrhizal fungi (EcMF), (Brundrett and Tedersoo, 2018). While AM are formed by one monophyletic group of fungi, the EcM lifestyle independently evolved multiple times within the fungal kingdom and can be found in various groups within the Ascomycota and Basidiomycota (Martin et al., 2016). In EcM, the fungus typically does not penetrate the plant cells but forms a dense, labyrinthine structure of hyphae between the epidermal and cortical plant root cells, called the Hartig net, and encloses the plant root with a hyphal sheath (Brundrett and Tedersoo, 2018). AM and EcM do not only differ morphologically, but also in their capabilities to take up nutrients, e.g., the mobilization of *N* and *P* from organic substrates (Read and Perez-Moreno, 2003).

Dual-mycorrhizal plants vary in the extent to which they are colonized by and depend on either of the two mycorrhizal types. In some species, both types can be found simultaneously within the same root system, while in others the presence of either type will depend on the life history stage of the host plant and local environmental factors such as soil conditions or the surrounding vegetation (Teste et al., 2020). In their recently proposed classification of dual-mycorrhizal plants, Teste et al. (2020) call the former context-free dual-mycorrhizal plants and the latter temporally dependent or spatially dependent dual-mycorrhizal plants. In temporally dependent dual-mycorrhizal plants, mycorrhizal type dominance varies between life history stages of a species, while in spatially dependent dual-mycorrhizal plants mycorrhizal type dominance varies between habitats or regions (Teste et al., 2020). Due to the eco-physiological differences between the two mycorrhizal types, the type that will be most beneficial largely depends on local habitat characteristics (Read and Perez-Moreno, 2003).

Surrounding vegetation is known to affect mycorrhizal community composition, by affecting local abiotic conditions, by providing the inoculum from which the roots are colonized or through competitive interactions between mycorrhizal types. Grünfeld et al. (2020), for example, showed that roots of AM forest herbs were more extensively colonized by arbuscular mycorrhizal fungi (AMF) in forest stands with a high cover of AM trees than in stands with a low cover of AM trees. McHugh and Gehring (2006) found that the presence of AM shrub negatively affected EcM colonization in *Pinus edulis*. How surrounding vegetation affects the interaction between EcMF and AMF within the same, dual-mycorrhizal plant species is however far less studied.

The major goal of this study was to gain a better understanding of how surrounding vegetation can affect the mycorrhizal communities of individual plants by testing the hypothesis that the mycorrhizal communities of the temperate tree *Crataegus monogyna* Jacq. strongly differ depending on the surrounding vegetation. Species from the genera *Alnus, Eucalyptus, Populus* and *Salix* are widely accepted to be dual-mycorrhizal, but for many other plant species the mycorrhizal status is not clear and under discussion (Brundrett and Tedersoo, 2020; Teste et al., 2020). This is caused by differences in definitions for the various mycorrhizal types, incorrect assignments of a certain mycorrhizal type and errors accumulating in databases [see Brundrett and Tedersoo (2020) and Teste et al. (2020) for more details]. One example of such a disputed plant species is *C. monogyna*, which is considered purely AM according to some sources (Brundrett and Tedersoo, 2020; Soudzilovskaia et al., 2020) and dual-mycorrhizal according to others (Trappe, 1962; Harley and Harley, 1987; Maremmani et al., 2003; Bueno et al., 2017; Teste et al., 2020). While its AM status is not under discussion, it is its EcM status that needs confirmation. More specifically, we assessed the dual-mycorrhizal nature of *C. monogyna* by microscopically examining roots for ectomycorrhizal diagnostic features (i.e., the presence of a Hartig net and hyphal sheath), and tested whether the mycorrhizal type of the surrounding vegetation (AM dominated grassland or mixed AM and EcM forest edge) affected the AMF and EcMF communities in the roots of *C. monogyna*.

## Materials and Methods

### Study Species

*Crataegus monogyna* Jacq. is a thorny shrub or small tree from the Rosaceae family, that occurs in deciduous forests, thickets and hedges. It is a common species within the study area (i.e., northeastern Belgium) and is native to most of Europe, North Africa and West Asia (Christensen, 1992). It is a broadleaved species that produces easily degradable litter and has some genus-specific associated saprotrophic fungi [e.g., *Tubaria dispersa* and *Parasola crataegi* (Szarkándi et al., 2017)]. It has a typical taproot system, with one well-developed taproot that grows vertically and several smaller lateral roots that grow horizontally (Kárász, 2006).

### Sampling

Root samples from twenty *C. monogyna* trees were taken in a paired sampling strategy across ten locations in central Belgium (Supplementary Figure 1). At each location two trees were selected, one in the forest edge, surrounded by vegetation of both the ectomycorrhizal (EcM) and arbuscular mycorrhizal (AM) type and one in grassland, surrounded purely by vegetation of the AM type. Sampling took place in the forest edge to minimize differences in soil conditions and light exposure between the samples from mixed EcM + AM vegetation and pure AM vegetation. The dominant EcM tree species in the forest edge was *Quercus robur* at each sampling location, sometimes intermixed with *Salix* spp. or *Corylus avellana*. Root samples were taken by following a large lateral root from the base of the stem until the finest roots at three points around the stem. Only the finest roots were collected and transported to the lab, where they were carefully washed to remove soil particles. From these washed roots, 0.2 g was used for DNA extraction and the rest to determine root colonization levels. Additionally, five soil cores were taken with a soil auger around the base of each sampled tree and pooled into one composite soil sample.

### Microscopic Analysis

To determine root colonization levels, root samples were cleared in 5% KOH at 65°C for 1.5 hours, washed with distilled water and subsequently stained in 0.05% Trypan blue at 65°C for 1.5 hours. Finally, they were transferred to lactoglycerol for destaining for 48 hours before they were microscopically examined. EcM root colonization levels were determined using the gridline intersections method under a dissecting microscope (Giovannetti and Mosse, 1980). When EcM root tips were found, sections were made from a subset of these root tips to check for EcM structures (Hartig net, mantle). Microscopic examination for the presence of AM fungi was done according to the magnified intersections method, as described by McGonigle et al. (1990). For each intersection the presence of arbuscules, vesicles, hyphal coils and aseptate hyphae was noted. Colonization levels of each of these structures were determined by dividing the number of intersections where these were found by the total number of intersections examined.

In one of the sampling locations, additional *C. monogyna* root samples were taken in the forest edge for more extensive root histology. Segments containing the root apex were fixed in 4% v/v paraformaldehyde in PEM buffer (50 mM piperazine-N,N′-bis(2-ethanesulfonic acid), 5 mM MgSO_4_ and 5 mM ethylene glycol tetraacetic acid, pH 6.9) for microscopic analysis, and the remaining segments were stored in 2× CTAB extraction buffer (2% w/v CTAB (Cetyl trimethylammonium bromide), 100 mM Tris-HCl, 1.4 M NaCl and 20 mM EDTA (Ethylene diamine tetraacetic acid) for molecular analysis. The samples were rinsed in phosphate-buffered saline (PBS: prepared from a 10× stock solution-−80 g NaCl, 28.6 g Na_2_HPO_4_.12H_2_O and 2 g KH_2_PO_4_ in 1 L demineralized H_2_O, pH 7.2), dehydrated in an increasing ethanol series (30, 50, 70, 85and 100% v/v), infiltrated with and embedded in LR White acrylate resin. Semi-thin (500 nm) sections were cut with a Leica Ultracut UC7 ultramicrotome equipped with a diamond histoknife (Diatome). Sections, collected on poly-L-lysine glass slides (Carl Roth), were stained with 1% w/v toluidine blue O and 1% w/v sodium tetraborate (Car Roth) and mounted in DPX (VWR).

A chitin-binding probe, wheat germ agglutinin (WGA) linked to fluorescein isothiocyanate (FITC; Sigma Aldrich) was used to stain hyphal cell walls. To this end, semi-thin sections were first incubated in 1 M NaOH for 2 hours to unmask chitin binding sites and then in 3% w/v milk protein to block non-specific binding sites. After rinsing with three changes of PBS for 10 minutes each, sections were incubated with 1:10 dilution of WGA-FITC in PBS for 2 hours. Negative control sections were prepared by omitting the antibody. Cellulose (plant cell walls) and chitin (hyphal cell walls) were stained with a beta-glucan-specific dye Calcofluor White M2R fluorochrome (Fluorescent brightener 28, Sigma, 0.25 μg ml-1 in dH_2_O) for 5 min. Sections were washed in PBS three times before mounting in a glycerol-based anti-fade solution (Citifluor AF2, Citifluor Ltd., UK). Slides were observed with a Nikon Ni-U epifluorescence microscope equipped with filter cubes for Calcofluor White (excitation 365/15BP; dichroic mirror 400LP; emission 420LP) and FITC (excitation 480/20BP; dichroic mirror 505LP; emission 410LP) imaging. Images were recorded using a Nikon DS-Fi3 digital camera.

### Molecular Analysis of Plant Root Identity

To confirm that the sampled roots belonged to *C. monogyna*, DNA was extracted from the root segments stored in 2× CTAB buffer using the DNeasy Plant mini kit (Qiagen) according to the manufacturer's instructions. Two regions were amplified: (1) the internal transcribed spacer (ITS) of nuclear ribosomal DNA using the primers ITS1 and ITS4 (White et al., 1990), (2) the intergenic spacer trnH-psbA of the plastid DNA using the primers psbAF and trnHR (Sang et al., 1997). Protocols for PCR amplification follow Le et al. (2007). PCR products were sequenced using an automated ABI 3,730 XL capillary sequencer at Macrogen. Forward and reverse sequences were assembled into contigs and edited where needed with BioloMICS (BioAware SA NV). The resulting sequences were blasted against NCBI GenBank using BLASTN with standard parameters.

### Metabarcoding

Root samples used in meta-barcoding were shredded before weighing 0.2 g for DNA extraction. DNA was extracted from these root samples and from 0.25 g of the soil samples using the Soil DNA Isolation Plus kit (Norgen Biotek Corp.) according to the manufacturer's instructions. From the DNA, two regions were amplified: the ITS2 rDNA region and part of the 18S SSU rDNA region. The first was amplified using the ITS86F and ITS4 primer pair, which mainly targets Dikarya fungi and is thus suitable to determine EcMF communities (Op De Beeck et al., 2014). The second region was amplified using the AMV4.5NF and AMDGR primer pair, which specifically targets Glomeromycota and is thus suitable to determine AMF communities (Van Geel et al., 2014). These primers were specifically modified to allow for metabarcoding on Illumina Miseq and consisted of the Illumina adaptor sequence, a unique index sequence, a 10 nucleotide pad sequence, a two nucleotide linker sequence and the gene-specific primer (Kozich et al., 2013). Amplification through PCR was carried out in 25 μl reaction volumes, each consisting of 1 μl of template DNA, 0.5 μl of each of the primers (20 μM), 5 μl ALLin HiFi Buffer (1.25 mM dNTPS, 15 mM MgCL_2_), (HighQu, Kraichtal, Germany) and 0.25 μl ALLin HiFi DNA Polymerase (2 u/μl), (HighQU, Kraichtal, Germany). PCR reactions with the ITS86F and ITS4 primer pair started with 1 min at 95°C, followed by 35 cycles of 20 sec at 95°C, 30 sec at 52°C and 30 sec at 72°C and ended with 5 min final extension at 72°C. PCR reactions with the AMV4.5NF and AMDGR primer pair started with 1 min at 95°C, followed by 35 cycles of 15 sec at 95°C, 45 sec at 65°C and 60 sec at 72°C and ended with a 5 min final extension at 72°C. PCR products were purified using Agencourt AMPure XP beads (Beckman Coulter, Brea, CA) and equimolarly pooled in two libraries (one for each primer pair) after measuring the concentration on the Qubit 3.0 Fluorometer (Invitrogen, NY, USA). The size distribution and quality of the library was checked using gel electrophoresis and DNA of the right size was extracted from the gel using QIAquick gel extraction kit (Qiagen). Finally, the libraries were diluted to 2 nm and sent to Genomics Core Leuven for sequencing on Illumina Miseq 2 × 250.

### Bioinformatics

For each of the two metabarcoding datasets, demultiplexed reads were merged, quality filtered and clustered into OTUs using USEARCH (Edgar, 2010). First, forward and reverse reads were merged using the *fastq_mergepairs* command with a maximum of 10 mismatches and a minimum 80% id of alignment. From these merged pairs, sequences shorter than 200 bp and with more than 1.0 expected errors were filtered out using the *fastq_filter* command. Using the *fastx_uniques* command, unique sequences were recovered to use in 97% Operational Taxonomic Unit (OTU) clustering with the *cluster_otus* command. Finally, an OTU table was constructed with the *otutab* command. To minimize the number of erroneous sequences, which can be produced during PCR and sequencing (Alberdi et al., 2018), an extra filtering step was performed for each sample in which we removed OTUs that were represented by <0.01% of the sequences in that sample. Reference sequences from the ITS2 dataset were compared against the UNITE database (Kõljalg et al., 2013) and sequences that could not be attributed a taxonomy with certainty of 0.8 or higher were BLASTed against NCBI GenBank with the following criteria for a hit: sequence similarity at least 97% (for species-level identification) or 90% (for genus-level identification), alignment at least 95% and *E*-value <1*e* – 50. To determine the ecological guild of all OTUs that could be identified at least at genus-level, FUNGuild was used (Nguyen et al., 2016). All OTUs with an ectomycorrhizal lifestyle were retained in one OTU table for further analyses (hereafter referred to as the EcMF OTU table). Consensus sequences from OTUs of the 18S SSU dataset were BLASTed against the MaarjAM database (Öpik et al., 2010) with the following criteria for a hit: sequence similarity at least 97%, alignment at least 95% and *E*-value <1*e* – 50. Sequences that did not return a hit were BLASTed against NCBI Genbank with the same criteria as for the ITS2 dataset. All OTUs belonging to the Glomeromycota were united in one OTU table for further analyses (hereafter referred to as the AMF OTU table). Raw sequencing data was submitted to the NCBI Sequence Read Archive and is available under Bioproject PRJNA672927.

### Soil Analyses

From the soil samples, soil pH, moisture content, organic matter content, and ammonium, nitrate and phosphorus concentration were determined. Soil pH was measured with a pH probe in a 1:5 soil: deionized water mixture which was first shaken for 20 min. Moisture and organic matter content of the soil were determined through the weight loss of ±10 g of soil after drying at 105°C for 12 hours and after combustion of organic material at 650°C for 2 hours. Soil ammonium and nitrate concentration were measured by shaking 5 g of soil in 25 ml of 1 M KCl for 30 min, centrifuging the solution for 5 min at 3,500 g and colometrically analyzing the supernatant on an Evolution 201 UV-visible spectrophotometer (Thermo Scientific, Madison, USA). Plant-available phosphorous was determined through the use of anion exchange resin membranes (BDH Chemicals Ltd. Poole England), which were first shaken in 0.5 M NaHCO_3_ for 16 hours, then shaken for 16 hours in a solution of 3 g soil in 30 ml deionized water and finally rinsed and shaken in 0.5 M HCl for 16 hours. The phosphorus concentration of this HCl solution is then determined by measuring the Malachite Green reaction with the Evolution 201 UV-visible spectrophotometer (Thermo Scientific, Madison, USA).

### Statistical Analyses

Differences in soil environmental factors (pH, moisture, organic matter, ammonium, nitrate and phosphorus) between the grassland and forest edge were tested using paired *t*-tests. Generalized Linear Mixed Models (GLMM) were used to test for differences in the number of EcMF sequences, AMF sequences, EcMF OTUs and AMF OTUs between vegetation types (grassland or forest edge) and sample types (root or soil). Sampling site was added as a random factor and either a Poisson or negative binomial distribution was used in the GLMMs. To test for differences in root colonization levels by arbuscules, aseptate hyphae, vesicles and hyphal coils between vegetation types, GLMMs were used with a binomial distribution and sampling site as a random factor. Models were fitted using the lme4 package (Bates et al., 2015). Redundancy analysis (RDA) was used to analyze patterns in community composition for samples with more than 100 sequences. The *ordiR2step* function from the *vegan* package (Oksanen et al., 2019) was used in forward stepwise selection of the best model, starting from the full model which included vegetation type (grassland or forest edge), sample type (root or soil), soil environmental factors, and spatial structure [through the use of principal coordinate analysis of neighbor matrices (PCNM), calculated with the *pcnm* function from the *vegan* package (Oksanen et al., 2019)]. Permutation tests with 999 permutations were used to test the significance of axes and terms of the final model. These analyses were performed twice, once with the AMF OTU table and once with the EcMF OTU table.

## Results

### Microscopic Analysis

**Figure 1:**
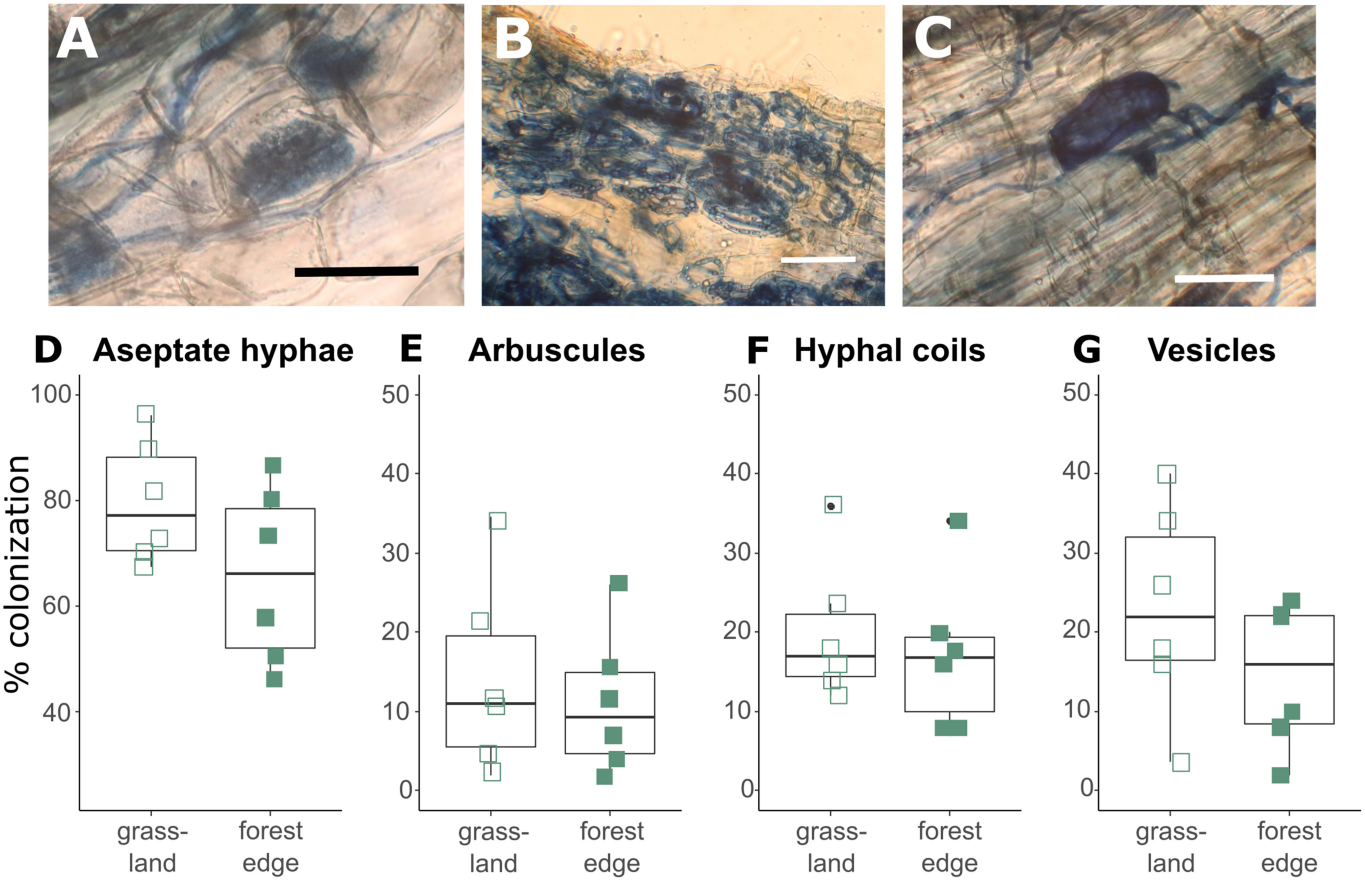
Arbuscular mycorrhizal structures found in the roots of *Crataegus monogyna* included aseptate hyphae, arbuscules **(A)**, hyphal coils **(B)** and vesicles **(C)**. Hyphal colonization rates **(D)** were significantly higher (GLMM: *z* = −2.239, *p* = 0.025) and arbuscular colonization rates **(E)** tended to be higher (GLMM: *z* = −1.774, *p* = 0.076) in root samples from grassland than from the forest edge. No significant difference was found in root colonization levels by hyphal coils **(F)** and vesicles **(G)** between root samples from grassland and from forest edge. Scale bar: 50 μm.

*Crataegus monogyna* roots were extensively colonized by AMF with hyphal colonization rates varying between 46 and 96%. Hyphal colonization rates were significantly lower (GLMM: *z* = −2.239, *p* = 0.025) in root samples from the forest edge (65.67 ± 16.61%) than in root samples from grassland (79.55 ± 11.70%). Arbuscular mycorrhizal structures included arbuscules (12.60 ± 10.34%, Figure 1A), hyphal coils (18.62 ± 8.90%, Figure 1B) and vesicles (18.82 ± 11.66%, Figure 1C), showing the co-occurrence of the *Paris*-type and *Arum*-type AM. Root colonization by arbuscules tended to be lower (GLMM: *z* = −1.774, *p* = 0.076) in root samples from the forest edge (11.11 ± 8.94%) than from grassland (14.09 ± 12.24%). There was no significant difference in colonization levels by hyphal coils or vesicles (Figures 1D–G). Ectomycorrhizal structures were found in four of the 12 samples studied microscopically, which all came from the forest edge. Ectomycorrhizal colonization levels were low however, varying between 5.7 and 9.6%.

**Figure 2:**
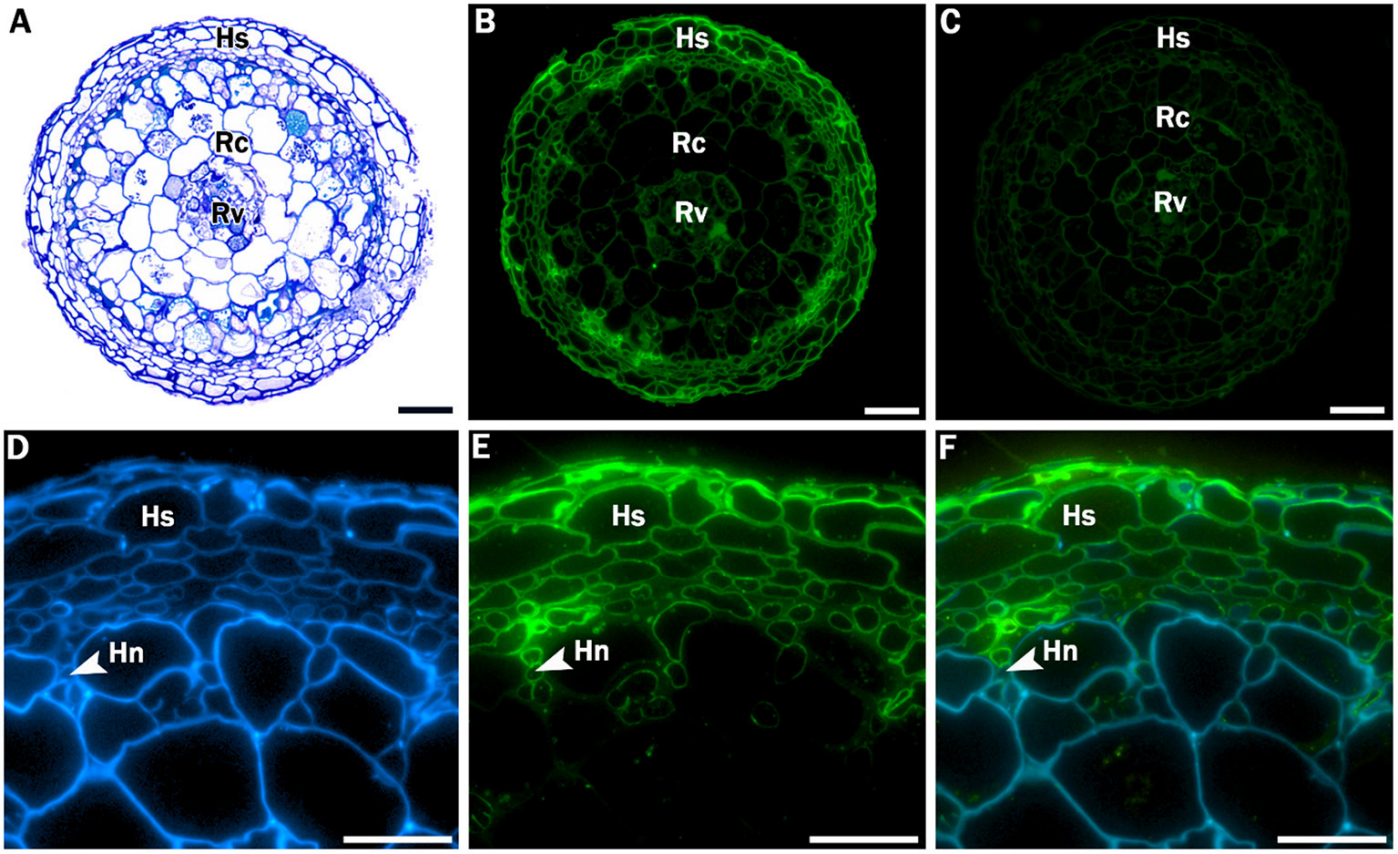
Stained and labeled transverse *Crataegus monogyna* root sections. **(A)** Overview section stained with toluidine blue O. **(B)** Equivalent section labeled with WGA-FITC; cell walls of the hyphal sheath are clearly labeled. **(C)** Equivalent control section showing weak auto fluorescence of plant root cell walls. **(D)** Detail of section labeled with calcofluor white, which stains both hyphal and plant cell walls. **(E)** Equivalent section labeled with WGA-FITC, which clearly binds to the cell walls of the hyphal sheath and the Hartig net. **(C)** Overlay of image **(D)** and **(E)**. Hs: hyphal sheath; Rc: root cortex; Rv: root vascular cylinder; Hn: Hartig Net; Scale bars: **(A-C)**: 100 μm; **(D-F)**: 40 μm.

Additional, more extensive root histology of a transverse section through a *Crataegus* root tip showed a hyphal sheath that completely surrounded the mycorrhizal root as well as a Hartig net, an intercellular network of hyphae that penetrates through the peripheral root cortical cell layers (Figure 2). To provide stronger evidence for the presence of these fungal structures, sections were incubated with WGA-FITC, which stains chitin in fungal cell walls, allowing discrimination between fungal and plant cell walls. WGA-FITC labeling was restricted to the structures that were morphologically identified as the hyphal sheath and Hartig net (Figures 2B,E,F).

Sequencing of the root segment originating from the same root sample used in microscopic analysis confirmed that the sampled roots belonged to *C. monogyna* (Supplementary Table 1). For the ITS sequence, the first hit corresponded with *C. monogyna*, with an *E*-value of 0.0 and a sequence similarity percentage of 99.84%. The second and third hit had the same sequence similarity percentage, but corresponded to *C. heldreichii*, a closely related species that does not naturally occur in the study area. For the intergenic spacer trnH-psbA sequence, the BLAST result showed a sequence similarity percentage of 100% and low *E*-values corresponding with *C. monogyna*. Combining the BLAST results from both molecular markers confirmed that the root tip used for the microscopic study unambiguously belonged to *C. monogyna*. The combination of the molecular and aforementioned morpho-chemical experiments therefore suggests that *C. monogyna* roots can engage in ectomycorrhizal associations.

### Soil Conditions

No significant differences in soil conditions were found between soil samples collected in grassland vegetation and samples from the forest edge (Supplementary Table 2). Grassland and forest edge soils had on average respectively a pH of 6.51 and 6.14, 15.8 and 15.9% moisture content, 12.2 and 12.8% soil organic matter content, 36.1 and 33.1 mg plant-available *P* per kg soil, 15.6 and 7.5 mg ${\text{NO}}_{3}^{-}$ per kg soil and 2.8 and 1.4 mg ${\text{NH}}_{4}^{+}$ per kg soil.

### Ectomycorrhizal Fungal Communities

**Figure 3:**
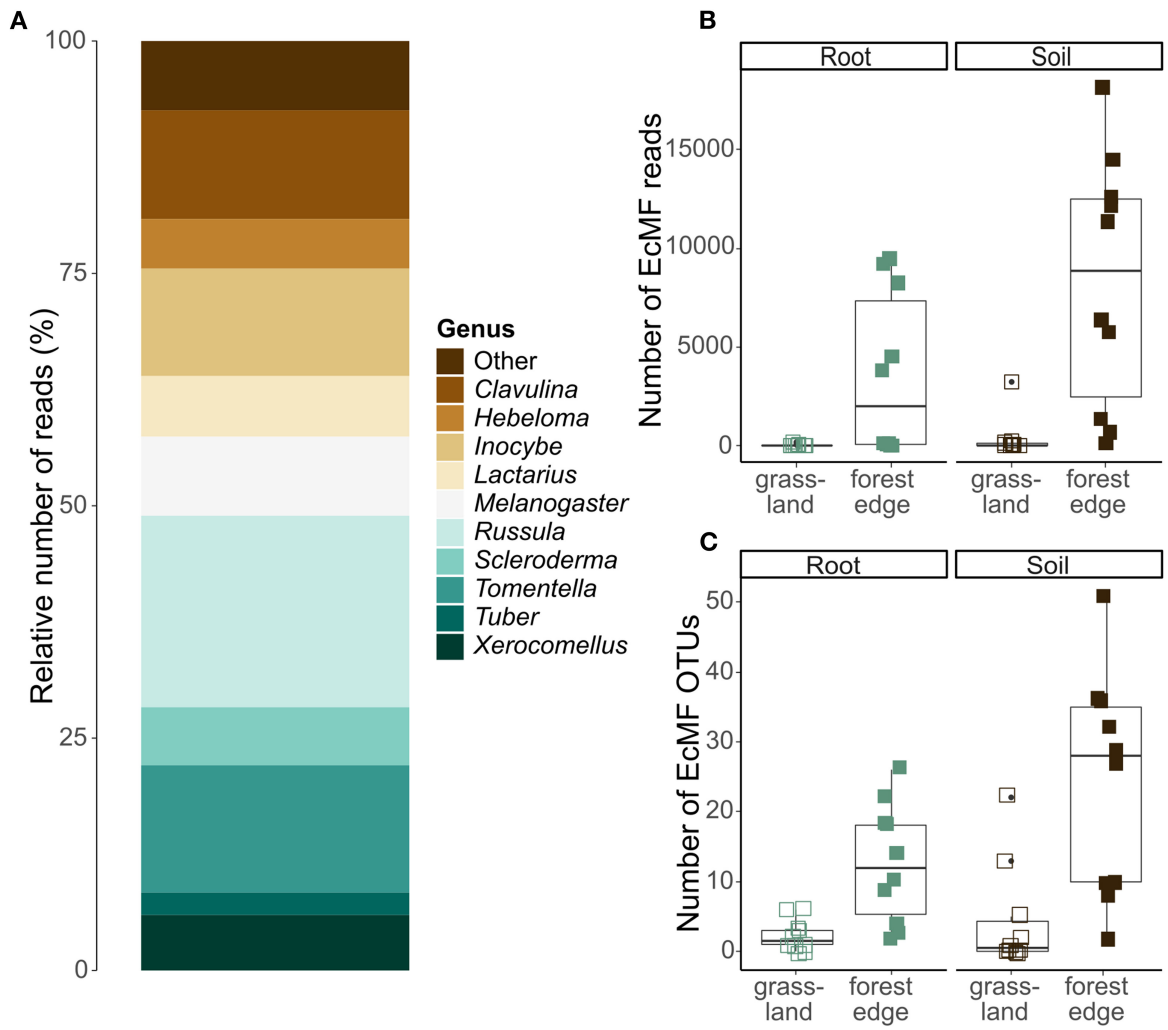
Ectomycorrhizal community composition and OTU diversity in the roots of *Crataegus monogyna* collected in grassland and forest edge vegetation as determined through metabarcoding with the ITS86F and ITS4 primer pair. **(A)** Relative number of sequences of EcMF genera. **(B)** The number of EcMF sequences was significantly higher in the mixed AM + EcM vegetation of the forest edge than in AM-dominated grassland vegetation and in soil samples compared to root samples. **(C)** The number of EcMF OTUs was significantly higher in samples from the forest edge compared to grassland and in soil samples compared to root samples.

After quality filtering and clustering of the ITS2 database, 879 036 sequences were assigned to 2,530 OTUs. Of those OTUs, 385 were assigned a taxonomy at genus-or species-level with a minimum certainty of 0.8 with the UNITE database. Additionally, 1,040 OTUs were assigned a taxonomy at genus-or species-level via NCBI Genbank, while the other 1,105 OTUs could not be reliably assigned to a genus. According to FUNGuild, 170 OTUs (122 631 sequences) had an ectomycorrhizal lifestyle, which were used in further analyses. These OTUs belonged to 33 genera, of which *Tomentella* (31 OTUs)*, Inocybe* (21 OTUs)*, Russula* (20 OTUs)*, Cortinarius* (16 OTUs) and *Hymenogaster* (11 OTUs) were the most OTU rich. Overall, most EcMF sequences belonged to the genus *Russula* (20.0% of the sequences), followed by *Tomentella* (13.2%), *Clavulina* (12.8%), *Inocybe* (11.5%) and *Melanogaster* (8.2%), (Figure 3A).

The number of EcMF sequences differed significantly between samples from grassland and forest edge (GLMM: *z* = 4.636; *p* < 0.001) and between root and soil samples (GLMM: *z* = 2.192, *p* = 0.028). Samples from the forest edge had more EcMF sequences than samples from grassland (respectively 5,933 ± 5,743 sequences and 199 ± 712 sequences). Soil samples contained on average 4,340 ± 6,013 sequences, while root samples contained 1,792 ± 3,350 sequences (Figure 3B). Similarly, EcMF OTU richness was significantly higher in samples from the forest edge than in samples from grassland (respectively 18 ± 14 OTUs and 3 ± 5 OTUs; GLMM: *z* = 12.9, *p* < 0.001) and in soil samples than in root samples (respectively 14 ± 16 OTUs and 7 ± 8 OTUs; GLMM: *z* = 6.4, *p* < 0.001), (Figure 3C). Sample type, however, had no significant effect on community composition. The best model explaining variation in EcMF community composition included soil pH, spatial variability (in the form of the first eigenvector of the principal coordinate analysis of neighbor matrices or PCNM) and soil moisture content (Supplementary Table 2A). Comparison of EcMF community composition between samples from forest edge and grassland was not possible due to too few samples from grassland with sufficient EcMF sequences.

### Arbuscular Mycorrhizal Fungal Communities

**Figure 4:**
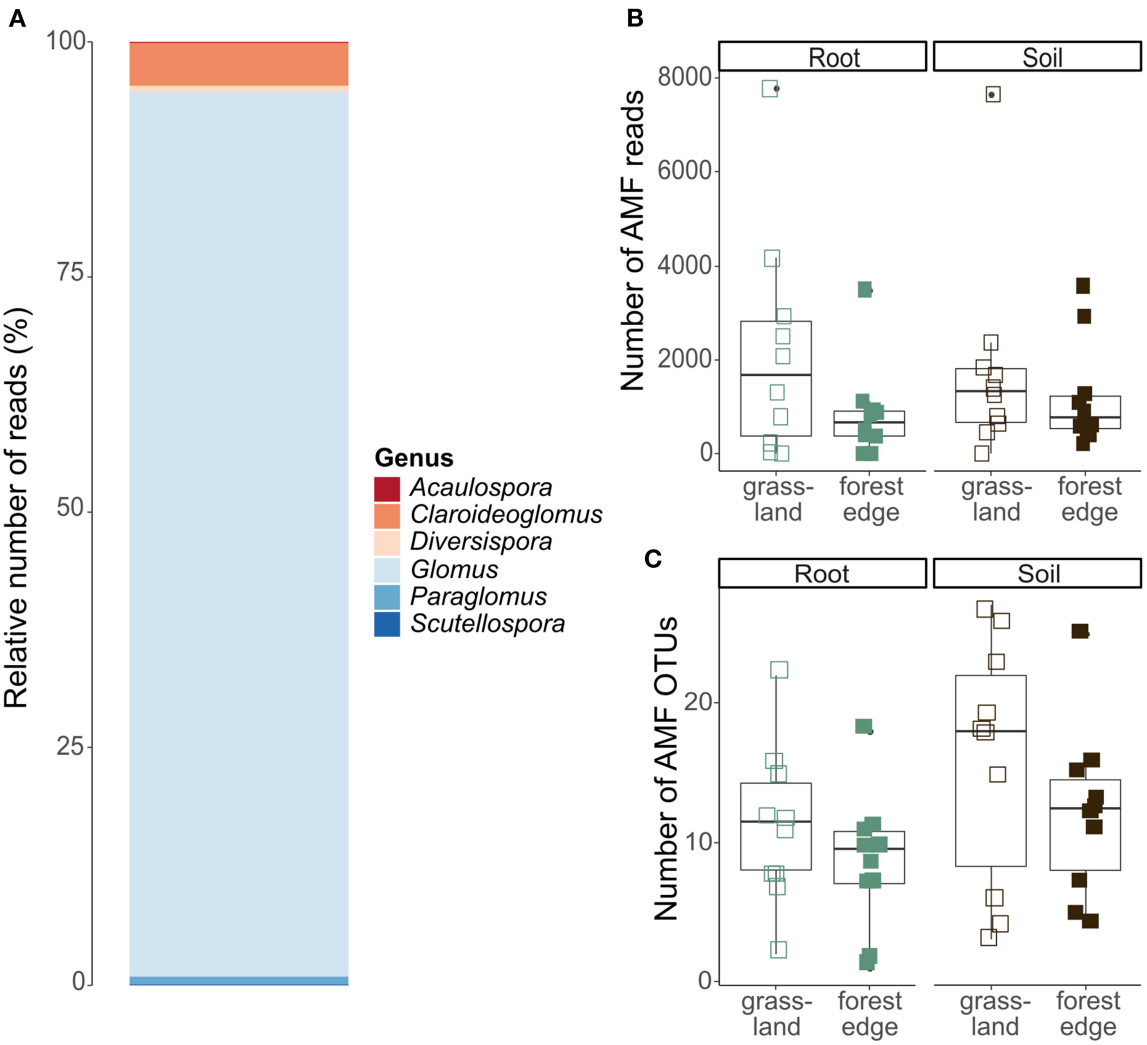
Arbuscular mycorrhizal community composition and OTU diversity in the roots of *Crataegus monogyna* collected in grassland and forest edge vegetation as determined through metabarcoding with the AMV4.5NF and AMDGR primer pair. **(A)** Relative number of sequences of AMF genera. **(B)** The number of AMF sequences did not differ between the mixed AM + EcM vegetation of the forest edge and the AM-dominated grassland vegetation or between soil and root samples. **(C)** AMF OTU richness was lower in samples from the forest edge compared to samples from grassland and in root samples compared to soil samples.

After quality filtering and clustering of the 18S SSU database, 427 099 sequences belonging to 1,127 OTUs remained. Of these, 60 466 sequences belonging to 83 OTUs could reliably be attributed to the Glomeromycota and were put together in the AMF OTU table. AMF communities were dominated by the genus *Glomus* (54 OTUs and 87.6% of the sequences), while the other genera *Claroideoglomus* (18 OTUs and 8.5% of the sequences)*, Paraglomus* (2 OTUs and 1.9% of the sequences)*, Scutellospora* (2 OTUs and 0.8% of the sequences)*, Diversispora* (2 OTUs and 0.8% of the sequences) and *Acaulospora* (5 OTUs and 0.4% of the sequences) were much less abundant (Figure 4A).

No significant difference in the number of AMF sequences was found between samples from grassland and from forest edge (GLMM: *z* = −1.70, *p* = 0.09) or between root and soil samples (GLMM: *z* = 0.21, *p* = 0.83), (Figure 4B). AMF OTU richness was, however, lower in samples from the forest edge compared to samples from grassland (respectively 10 ± 6 OTUs and 14 ± 8 OTUs; GLMM: *z* = −2.97, *p* = 0.003) and in root samples compared to soil samples (respectively 10 ± 5 OTUs and 14 ± 8 OTUs; GLMM: *z* = 3.69, *p* < 0.001), (Figure 4C). AMF community composition did not differ between soil and root samples or between samples from forest edge and from grassland. The best RDA model explaining variation in AMF community composition included soil pH and phosphorus (Supplementary Table 2B).

## Discussion

### Is *Crataegus monogyna* a Dual-Mycorrhizal Plant?

The presence of a Hartig net and hyphal sheath in and around the roots of *C. monogyna* indicates that this species is capable of forming ectomycorrhizal (EcM). Some saprotrophic fungi have been found to show affinity for roots and to form mantle-like structures (Smith et al., 2017), but metabarcoding of the root-associated fungal communities demonstrated the presence of typical EcMF taxa in *C. monogyna* roots.

However, root colonization levels were low and EcM structures were only found in samples from the mixed AM + EcM vegetation, suggesting that *C. monogyna* is not able to independently support EcM fungi. Whether *C. monogyna* can be considered a dual-mycorrhizal species consequently depends on the definition of an EcM plant: whether it is a species capable of forming EcM structures or a species capable of supporting EcMF (Teste et al., 2020). These results also raise the question whether other species of the genus *Crataegus* are able to form ectomycorrhizal. Although closely related species often share mycorrhizal types or nutritional strategies, this is less often the case in this type of flexible mycorrhizal associations where the mycorrhizal type depends on environmental circumstances (Gerz et al., 2018). Simply attributing the same mycorrhizal status to all other *Crataegus* species will thus probably result in misclassification errors (Bueno et al., 2019). On the other hand, it is likely that *Crataegus* species that are more typically found in forests, such as *C. laevigata* or *C. mollis*, also form ectomycorrhizal and it would thus be interesting to search for EcM structures in these species.

### Variation in Mycorrhizal Type Dominance Is Dependent on Vegetation Type

Surrounding vegetation is known to affect mycorrhizal root colonization and mycorrhizal community composition, both in AMF (Hausmann and Hawkes, 2009; Grünfeld et al., 2020) and EcMF (Dickie et al., 2004; Hubert and Gehring, 2008). This effect is mostly attributed to the increased availability of inoculum with increasing presence of plants of a certain mycorrhizal type and to host preferences of mycorrhizal fungi (Ishida et al., 2007). Here, we found a much lower number of EcMF sequences and OTUs in the soil of grasslands, indicating a much lower EcMF inoculum availability. In contrast, higher OTU diversity and sequence numbers were found in samples collected along forest edges. EcM structures were also only found in root samples collected in the forest edge. AMF OTU richness, hyphal and arbuscular root colonization, on the other hand, were higher in samples from grassland than from the forest edge. These results indicate that the mycorrhizal type of the surrounding vegetation can have a pronounced effect on the presence of a mycorrhizal type. This has already been observed in tree seedlings, especially after disturbances. For example, Dickie et al. (2001) showed that *Quercus rubra* seedlings planted near *Acer* (AM) stumps in a logged forest stand had higher AMF root colonization rates than seedlings planted near *Quercus* stumps, which had the highest EcMF colonization rate. In another study, AMF were more frequently encountered on *Pinus muricata* seedlings that established in AM-dominated scrub than in EcM-dominated forest after wildfire (Horton et al., 1998). Colonization of these seedlings by EcMF took longer, but once these fungi had colonized the roots, they were more diverse in the EcM-dominated forest than in the AM-dominated scrub where EcMF inoculum availability was much lower.

*C. monogyna* roots were extensively colonized by AMF, forming both the *Arum*-type and the *Paris*-type. Both morphological types have been found in the Rosaceae family before but not simultaneously in the same species (Dickson et al., 2007). But the co-occurrence of the two types is known occur in other plant species (Kubota et al., 2005; Salomón et al., 2014). While the presence of AM in *C. monogyna* roots is standard, the low colonization rates by EcM structures suggest it is optional. To what extent *C. monogyna* and its mycorrhizal partners benefit from the dual colonization remains unknown. Flexibility in mycorrhizal associations has been found to correlate with niche breadth (Gerz et al., 2018). It is possible that optional association with EcMF increases the niche *C. monogyna* can occupy, e.g., through increased flexibility throughout ecosystem development. *C. monogyna* can facilitate the natural succession from grassland to forest by increasing seedling survival of late-successional, shade-tolerant tree species (Gómez-Aparicio et al., 2004). It is also possible that associating with EcMF increases the flexibility of *C. monogyna* to cope with changes in soil properties (e.g., soil temperature, litter type), soil microbial communities and/or surrounding vegetation during forest succession (Teste et al., 2020).

### Effects of Local Soil Conditions?

Although the dominant mycorrhizal type is known to affect soil conditions (Tedersoo and Bahram, 2019), no significant difference in soil conditions was found between samples collected from AM dominated grassland and from mixed AM + EcM forest edge. This result indicates that the observed differences in EcM presence can be attributed to differences in inoculum availability and are not the result of differences in soil pH or nutrient availability as a cause. Both EcMF and AMF communities were affected by local soil conditions (respectively soil pH and moisture and soil pH and plant-available phosphorus). This is in line with other studies that have shown that abiotic conditions are important in structuring mycorrhizal communities (Boeraeve et al., 2018, 2019; van der Linde et al., 2018; Van Geel et al., 2018).

Our results further showed that EcMF and AMF community composition did not significantly differ between soil and root samples, suggesting that *C. monogyna* associates with a random selection of whatever is present in the soil surrounding its root system. Although EcM plants generally associate with a broad range of EcMF, most EcMF show at least some host specificity toward or preference for a particular host plant and EcM plants thus often differ in their EcMF communities, even when growing together (Bruns et al., 2002; Ishida et al., 2007; Lang et al., 2011). In contrast, AMF are considered to have a low host specificity, but some studies have found moderate host selectivity in both grasslands and forests (Öpik et al., 2009; Sepp et al., 2019). The fact that no differences in mycorrhizal community compositions were found between root and soil samples could be an indication that EcMF colonization of *C. monogyna* roots is due to opportunistic behavior of the tree, the EcMF or both.

## Conclusion

Overall, we conclude that *C. monogyna* is a tree species that is capable of forming associations with fungi that form ectomycorrhizal and arbuscular mycorrhiza. While the species consistently associated with AM, its association with EcM depended on the surrounding vegetation and EcM colonization of the roots is rather low, suggesting *C. monogyna* is not capable of independently supporting EcMF. Whether it can be considered a dual-mycorrhizal plant species thus depends on how an EcM plant is defined: based on morphology (the presence of EcM structures) or functionality (the mutualistic association with EcMF). Further research is needed to determine whether other species of genus *Crataegus* have the same properties and whether *C. monogyna* and/or its mycorrhizal partners experience benefits from dual colonization of the roots.

## Data Availability Statement

The datasets presented in this study can be found in online repositories. The names of the repository/repositories and accession number(s) can be found below: https://www.ncbi.nlm.nih.gov/genbank/, PRJNA672927.

## Author Contributions

All authors participated in the conception, and design of the study. MB performed the sampling, soil chemical analyses, bioinformatics, statistical analyses, and wrote the first draft of manuscript. MB, OL, and RD performed the microscopic analyses. All authors contributed to previous versions of the manuscript.

## Funding

The research was funded by the KU Leuven (CELSA/19/015).

## Conflict of Interest

The authors declare that the research was conducted in the absence of any commercial or financial relationships that could be construed as a potential conflict of interest.

## Publisher's Note

All claims expressed in this article are solely those of the authors and do not necessarily represent those of their affiliated organizations, or those of the publisher, the editors and the reviewers. Any product that may be evaluated in this article, or claim that may be made by its manufacturer, is not guaranteed or endorsed by the publisher.
