# Supplementary material for: The Effect of Surrounding Vegetation on the Mycorrhizal Fungal Communities of the Temperate Tree Crataegus monogyna Jacq
Source: Front Fungal Biol. 2021 Oct 25;2:741813. doi: 10.3389/ffunb.2021.741813 (PMC10512229; doi:10.3389/ffunb.2021.741813)
Supplement: Supplementary file 1 [file Data_Sheet_1.PDF]

Supplementary Material

1 Supplementary tables

Table S1 Sequence-matching data of the root sample used for histological evaluation of ectomycorrhizal structures. For each sequenced region, the ten best matches against Genbank are given, together with accession numbers, max. and total score, query cover, e values and percentage identity.

ITS sequence
ggaaggatcattgtcgaacctgcgcagcagaacgacccgagaaccagtttcaacgccggggccccggcgggccttcgggctcggcgtcccctcgcgtcccgggagcccgctcccgggcgcacaaaacgaacaccggcgcgttttgcgccaaggaacctgaacgaaagaccgcgctcccgacgccccggaaacggtgcgcgcgcgggcgcgtcgtcgtcttcgatatatgtcaaaacgactctcggcaacggatatctcggctctcgcatcgatgaagaacgtagcgaaatgcgatacttggtgtgaattgcagaatcccgtgaaccatcgagtctttgaacgcaacttgcgcccgaagcctctaggccgagggcacgcctgcctgggcgtcacgcgccgttgcccccccctcgcctctctcgggagcgtcggcgggcggacgatggcctcccgtgcgccaccccgcgcggttggcccaaatgtcgagtccccggcgacgaacgccacgacaatcggtggttgtcaaacctcggttgcctgttgtgcgctttcgccgcgctgcgggcggctcgcgaccatcgcggctctgcttcggcggagctcttcaacgcg

Species	Accession nr.	Max Score	Total Score	Query Cover	E value	Per. ident
Crataegus monogyna	MT937116	1123	1123	100%	0.0	99.84%
Crataegus heldreichii	EU500465	1123	1123	100%	0.0	99.84%
Crataegus heldreichii	EF127016	1123	1123	100%	0.0	99.84%
Crataegus azarolus	MT113325	1118	1118	100%	0.0	99.67%
Crataegus laevigata	EU785942	1118	1118	100%	0.0	99.67%
Crataegus monogyna	EF127014	1116	1116	100%	0.0	99.51%
Crataegus laevigata	KJ506855	1114	1114	100%	0.0	99.51%
Crataegus laevigata	EU500466	1112	1112	100%	0.0	99.51%
Crataegus laevigata	EF127015	1112	1112	100%	0.0	99.51%
Crataegus babakhanloui	JN873307	1109	1109	100%	0.0	99.35%

intergenic spacer trnH-psbA sequence
ataatttccctctagacctagctgctgttgaagttccatctataaatggataagactttggtcttaatatatatgagttcttgaaagtaaagga gtaataaaaaatttcttgttatatcaagaggttttttattgctcctttactatttagttttagtatttttttagttattacttaactatttgtttttgttt tatttgttttattcttttttttttagtaaaaaaaaaaaaaagaataaagttttcaatttaggttgtattttatcttacaagtaatgctaaatggcgga aaaattattaatttgtaatactacagggggcggatgtagccaagtggatcaaggcagtggattgtgaatccaccatg

Description	Accession nr.	Max Score	Total Score	Query Cover	E value	Per. ident
Crataegus monogyna	FN687519	680	680	100%	0.0	100.00%
Crataegus pinnatifida	KY419945	651	651	100%	0.0	98.39%
Crataegus monogyna	MF348670	645	645	94%	0.0	100.00%
Crataegus monogyna	FJ493304	636	636	100%	3.00E-178	97.60%
Crataegus monogyna	AJ853465	632	632	92%	4.00E-177	100.00%
Crataegus sinaica	AJ853467	627	627	92%	2.00E-175	99.71%
Crataegus sp.	MK920294	623	623	100%	2.00E-174	97.06%
Crataegus sheila-phippsiae	KP050220	621	621	99%	8.00E-174	97.30%
Crataegus mollis	KP050215	621	621	99%	8.00E-174	97.30%
Crataegus brazoria	KP050204	621	621	99%	8.00E-174	97.30%

Table S2 None of the measured soil variables differed significantly between the sampling points in grassland and in the forest edge, as tested with paired t-tests.

Soil variable	t value	Degrees of freedom	p value
Soil pH	1.215	9	0.255
NH_4^+	0.799	9	0.445
NO_3^-	1.055	9	0.319
Moisture	-0.117	9	0.909
Soil Organic Matter	-0.521	9	0.615
Phosphorus	0.419	9	0.685

2 Supplementary Figures

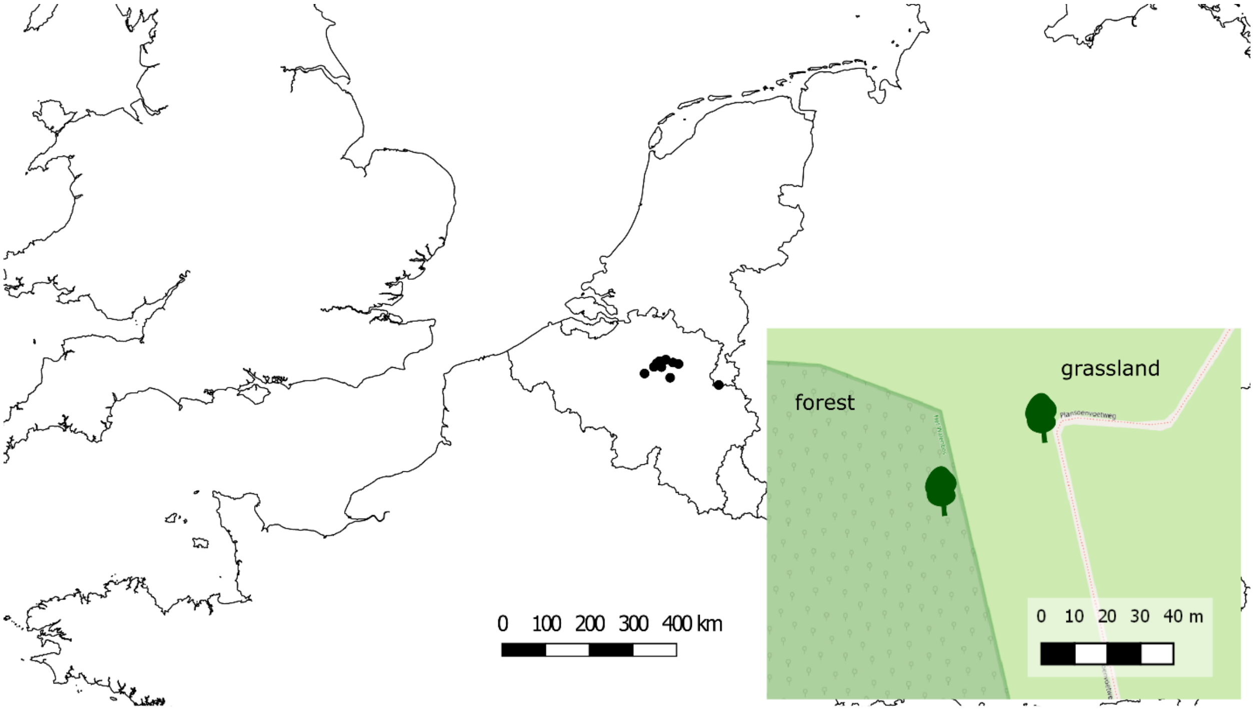

Supplementary Figure 1. Overview of sampling locations. In each location two *Crataegus monogyna* trees were sampled, one in grassland vegetation (dominated by arbuscular mycorrhizal plants) and one in the forest edge (surrounded by both ectomycorrhizal (EM) and AM plants). These were located at least 25m from each other (an example is shown in the insert).

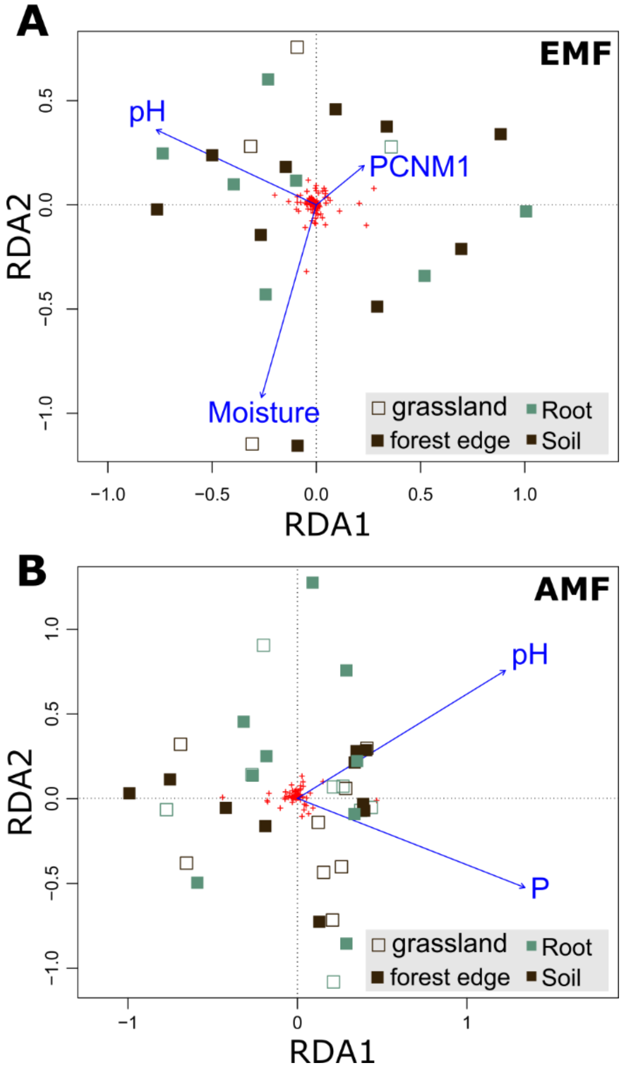

Supplementary Figure 2. The best RDA models explaining variation in community composition of EMF and AMF. A. Variation in EMF community composition was best explained by a combination of soil variables pH and moisture and spatial variability in the form of eigenvector 1 of the principal coordinate analysis of neighbor matrices (PCNM1). B. Variation in AMF community composition was best explained by soil pH and phosphorus content. Only samples with at least 100 sequences were used in this analysis (EMF: n=21, AMF: n=35).
